# Maternal vitamin A and D status in second and third trimester of pregnancy and bone mineral content in offspring at nine years of age

**DOI:** 10.3389/fendo.2024.1417656

**Published:** 2024-06-28

**Authors:** Astrid Kamilla Stunes, Mats Peder Mosti, Miriam Katarina Gustafsson, Torunn Børsting, Per Medbøe Thorsby, Signe Nilssen Stafne, Unni Syversen

**Affiliations:** ^1^ Department of Clinical and Molecular Medicine, Faculty of Medicine and Health Sciences, Norwegian University of Science and Technology (NTNU), Trondheim, Norway; ^2^ Center for Oral Health Services and Research, Mid-Norway (TkMidt), Trondheim, Norway; ^3^ Department of Research and Development, Clinic of Substance Use and Addiction Medicine, St. Olavs University Hospital, Trondheim, Norway; ^4^ Department of Public Health and Nursing, Norwegian University of Science and Technology (NTNU), Trondheim, Norway; ^5^ Hormone Laboratory, Department of Medical Biochemistry, Oslo University Hospital, Oslo, Norway; ^6^ Institute of Clinical Medicine, Faculty of Medicine, University of Oslo, Oslo, Norway; ^7^ Clinic of Rehabilitation, St. Olavs University Hospital, Trondheim, Norway; ^8^ Department of Endocrinology, Clinic of Medicine, St. Olavs University Hospital, Trondheim, Norway

**Keywords:** vitamin A, vitamin D, pregnancy, offspring bone mass, retinol, 25(OH)D, 1,25(OH)2D

## Abstract

**Introduction:**

Maternal nutritional and vitamin status during pregnancy may have long-term effects on offspring health and disease. The aim of this study was to examine the associations between maternal vitamin A and D status in pregnancy and offspring bone mineral content (BMC) at nine years of age.

**Methods:**

This is a *post-hoc* study of a randomized control trial including 855 pregnant women from two Norwegian cities; Trondheim and Stavanger. The women were randomized into an exercise intervention or standard antenatal care. Mother and child pairs for the present study were recruited from those still living in Trondheim after 8–10 years. Serum vitamin A (retinol) and vitamin D (25(OH)D) were measured in the 2^nd^ and 3^rd^ trimesters of pregnancy, and active vitamin D (1,25(OH)_2_D) in serum was measured in a subgroup. Spine BMC and trabecular bone score were measured in the children at nine years of age. Associations were analyzed with linear regression models.

**Results:**

A total of 119 mother and child pairs were included in the analyses. Vitamin A insufficiency (retinol**<** 1.05 µmol/L) and vitamin D deficiency (25(OH)D**<** 50 mmol/L) increased from ~7% to ~43% and from ~28% to ~33%, respectively, from the 2^nd^ to the 3^rd^ trimester. An increase in serum 1,25(OH)_2_D from the 2^nd^ to the 3^rd^ trimester was observed in the subgroup. There was a negative association between serum retinol in the 2^nd^ trimester and spine BMC in the boys, but not in the girls, when adjusted for maternal and child confounders. No other associations between maternal serum vitamin A or D and BMC in the children were found.

**Conclusion:**

We observed a high prevalence of vitamin A insufficiency and vitamin D deficiency during pregnancy. A negative association between mid-pregnancy vitamin A status and spine BMC was observed in boys, but not girls, while no associations were found between maternal vitamin D status and child BMC. The implications of optimal vitamin A and D status in pregnancy for offspring bone health, remains a subject for further investigations.

## Introduction

Environmental exposures in fetal and early life may permanently influence the vulnerability to disease later in life (1). The nutritional status of the mother during pregnancy is therefore of significance for the future health of the offspring. The *in utero* environment and access to nutritional factors, such as vitamin A and D, are important for the child’s future bone health and risk for osteoporosis (2–4).

Vitamin A is crucial for development of the skeleton at an early stage of embryogenesis, and both deficiency and excess can cause skeletal defects in the fetus (5). The growth of long bones and skeletal structural development in mid- and late pregnancy are dependent on access to vitamin A (2, 6). Vitamin D is pivotal for the mineralization of the fetal skeleton. A rise in the active form of vitamin D, 1,25-dihydroxyvitamin D (1,25(OH)_2_D), occurs through the second and third trimester of pregnancy, which has been proposed to optimize calcium absorption and promote accrual of calcium in the fetal skeleton (7, 8). Calcium, phosphorus, and magnesium are actively transported from the maternal circulation across the placenta, and most of this transfer occurs in the third trimester (9). The human fetus requires accretion of approximately 30 g of calcium during the 3^rd^ trimester for adequate bone development (10, 11).

According to the World Health Organization (WHO), vitamin A deficiency (VAD) defined as circulating retinol< 0.70 µmol/L (12), is still considered a public health problem. Deficiency is particularly prevalent in several developing countries, and around 20 million pregnant women are considered VAD globally (12–14). Vitamin A exists in two dietary forms, retinol and provitamin A (mainly β-carotene). Vitamin A is stored in the liver and transported in the circulation bound to retinol-binding proteins (15). After conversion to the active metabolite, all-trans-retinoic acid (ATRA), it binds to the retinoic acid receptor (RAR) and retinoic X receptor (RXR) heterodimers in the target tissue (16). Vitamin A intake is generally assumed to be sufficient in western populations. However, a study among pregnant women from USA published in 2019, reported that 60% had insufficient serum levels of retinol in the third trimester (17).

Vitamin D deficiency (VDD, serum 25(OH)D< 50 nmol/L) during pregnancy, is highly prevalent and considered a worldwide epidemic. A prevalence of VDD in pregnancy up to 90% is reported (18), and VDD is associated with adverse maternal, fetal, and neonatal outcomes (18, 19). Vitamin D is acquired through the diet or formed in the skin from 7-dehydrocholesterol under the influence of ultraviolet B radiation (UVB). Thereafter, vitamin D is converted to hydroxyvitamin D (25(OH)D) which is used to assess vitamin D status in the circulation. 25(OH)D is a precursor of the active form, 1,25(OH)_2_D, which binds to the vitamin D receptor by heterodimerizing with RXR (20). There is some controversy concerning the definition of VDD. According to the European Food Safety Authority (EFSA), vitamin D deficiency is defined as serum 25(OH)D levels< 30 nmol/L (21), while the Endocrine Society consider serum levels< 50 nmol/L as VDD (22).

Few studies have reported on associations between maternal vitamin A status and bone parameters in children. A study from the UK published in 2016 reported a negative association between maternal retinol serum concentrations in late pregnancy and whole body bone mineral content (BMC) in neonates, whereas maternal serum β-carotene concentrations were positively associated (23). On the other hand, we have previously reported a positive association between maternal serum retinol status and peak bone mass in adult offspring (24).

Studies on the association between maternal 25(OH)D concentrations during pregnancy and offspring BMC measured by dual x-ray absorptiometry (DXA), show conflicting results. Positive (25–27), or no (24, 28, 29) associations between maternal vitamin D status and offspring BMC at ages ranging from 6–26 years, are reported.

The objective of the current study was to investigate the relationship between maternal serum status of vitamin A (retinol) and vitamin D (25(OH)D) in the 2^nd^ and 3^rd^ trimesters of pregnancy and spine BMC and trabecular bone score (TBS), a measure of bone quality, in the children at nine years of age.

## Methods

### Study design, setting, and participants

In this longitudinal study, we used maternal and neonatal data collected from the randomized controlled trial (RCT) “Training in Pregnancy” (TRIP), conducted in Norway between 2007 and 2009, with the original primary aim to assess the effect of exercise for prevention of gestational diabetes (30). The original study included 855 pregnant women from two Norwegian cities (Trondheim and Stavanger), and the participants were randomized to a 12-week moderate-intensity exercise program or to standard prenatal care (30). Data and serum samples were collected in the 2^nd^ and 3^rd^ trimesters of the pregnancy (gestational weeks 18-22 and 32-36, respectively) (30). The mothers and their children who were still living in the Trondheim area, were invited to participate in a follow-up study when the children were 8 to 10 years of age. Data on the children were collected through questionnaires, blood sampling, anthropometrics, and dual X-ray absorptiometry (DXA) measurements at St. Olav’s University Hospital, Trondheim, Norway, between November 2016 and December 2018. Since participants from the two original study groups (30) were homogenous regarding all predictors included in the current study, both at inclusion and after the intervention, we merged all the participants into one group for the current analyses.

### Characteristics of the mothers

Data regarding the mothers’ age at pregnancy, education, smoking, pre-pregnancy body mass index (BMI, kg/m^2^), height, weight and BMI at inclusion (2^nd^ trimester), number of days per week with regular exercise training and if they participated in regular exercise training with high intensity three or more times per week, parity, sample season and children’s birthweight, gestational age, and mode of delivery were retrieved from the original study (30, 31). Data regarding intake of vitamin A, D and calcium from diet and supplements in the 2^nd^ and 3^rd^ trimesters, were extracted from a 180-items Food Frequency Questionnaire (FFQ) answered by the mothers at the two gestational measure points (32). Vitamin A intake is reported as Retinol Activity Equivalents (RAE), calculated as total retinol plus one twelfth of the β-carotene intake (33).

### Characteristics of children and DXA measurements

Height (cm) and weight (kg), without shoes and outer clothes, were recorded using a wall-mounted digital stadiometer, and BMI (kg/m^2^) was calculated. Iso-BMI, i.e. categories for BMI adjusted for age and sex, was obtained with the use of a calculator published by The Norwegian Institute of Public Health (34). BMC and bone mineral density (BMD) at the lumbar spine (L1-L4), whole body less head, distal femur and total hip, were measured by DXA, applying Hologic Discovery A S/N 83817. Children were asked to remove metal objects before the DXA scanning and carefully instructed to lie still to prevent movement artefacts. Spine TBS was calculated from spine BMD, weight, and height, using the TBS iNsight^®^ Software version 1.8 (Med-Imaps, Pessac, France). Some of the characteristics of these children have previously been published (35, 36).

### Serum analyses in the mothers

In the original TRIP-study, overnight fasting blood samples were collected from the pregnant women by standard venipuncture at inclusion in the 2^nd^ trimester and after ~14 weeks in the 3^rd^ trimester, as previously described (30, 31). Briefly, blood was collected in vacuum tubes, sat for 30 minutes at RT, centrifuged (3000g/4°C/10 minutes) before sera were aliquoted and stored at -80°C until further analyses. 25(OH)D) was analyzed by an electrochemiluminescence immunoassay (ECLIA), (Roche Cat# 09038078, RRID: AB_2909604), vitamin A (retinol) by high performance liquid chromatography (HPLC), intact parathyroid hormone (PTH) by ECLIA (Roche Cat# 07251068190 or 07251068500, RRID: AB_2895653), calcium and albumin by colorimetric methods, at the Department of Biochemistry, St. Olavs University Hospital, Trondheim, Norway. Vitamin D binding protein (DBP) was analyzed by an in-house competitive radioimmunoassay (RIA) (Sigma-Aldrich Cat# HPA019855, RRID: AB_1849545) and 1,25(OH)_2_D by an enzyme immunoassay (Immunodiagnostic Systems Cat# AC-62F1, RRID: AB_2891249) at the Hormone Laboratory, Oslo University Hospital, Oslo, Norway.

### Serum analyses in the children

At follow-up, overnight fasting blood samples were collected from the children (between 07:30 and 09:00). Children received a numbing Emla-patch containing a gel with lidocain and prilocaine 30 minutes before blood samples were collected by standard venipuncture. Blood was collected in vacuum tubes, sat for 30 minutes at RT, centrifuged (3000g/4°C/10 minutes), and sera were aliquoted and stored at -80°C until further analyses. Sera from all participants were analyzed simultaneously for 25(OH)D by a Liquid Chromatography with Tandem Mass Spectrometry (LC-MS/MS) method with DEQAS quality control (www.deqas.org) and intact parathyroid hormone (PTH) by a non-competitive immunoluminometric assay (ILMA) with an Immulite 2000 Intact PTH Kit (Siemens Cat# L2KPP-15, RRID: AB_2782968s) at the Hormone Laboratory, Oslo University Hospital, in March 2021. Sera from all children were analyzed simultaneously for albumin and total calcium at the Department of Biochemistry, St. Olavs University Hospital in September 2021.

In the current study we used clinical cut-offs based on serum measurements from the European Food Safety Authority (EFSA) for categorizing vitamin A deficiency (sRetinol< 0.7 µmol/L), insufficiency (sRetinol: 0.7-1.05) and sufficiency (sRetinol > 1.05 µmol/L) for the maternal measurements (37). We categorized extreme vitamin D deficiency (s25(OH)D< 30 nmol/L), deficiency (s25(OH)D: 30-50 nmol/L) and sufficiency (s25(OH)D ≥ 50 nmol/L) for both the mothers and the children, according to the recommendations from the Endocrine Society (22). Sample seasons were defined as winter and spring (December to May) and summer and autumn (June to November).

### Calculation of free and bioavailable 25(OH)D

The concentration of free 25(OH)D [D_free_] was calculated with the use of a binding constant between albumin and 25(OH)D (binding C_albumin-D_) equal to 6 x 10^5^ M^-1^, and a binding constant between DBP and 25(OH)D (binding C_DBP-D_) equal to 7 x 10^8^ M^-1^ (38):


$$\left[{\text{D}}_{\text{free}}\right]=\frac{\left[{\text{D}}_{\text{total}}\right]}{\left(1+\left(\left({\text{binding C}}_{\text{albumin}-\text{D}}\right)\times \left[\text{albumin}\right]\right)+\left({\text{binding C}}_{\text{DBP}-\text{D}}\right)\times \left[\text{DBP}\right]\right))\;}$$

The concentration of albumin-bound 25(OH)D [D_albumin_] was calculated as:


$$[{\text{D}}_{\text{albumin}}]=[{\text{D}}_{\text{free}}]\times {\text{binding C}}_{\text{albumin}-\text{D}}\times \left[\text{albumin}\right]$$

Bioavailable 25(OH)D was calculated as the total of free and albumin-bound 25(OH)D (39).

### Ethics

The study was conducted in accordance with the ethical principles in the Declaration of Helsinki. Ethical approval from the Norwegian Regional Committees for Medical and Health Research (REK) was given for the original TRIP-study (4.2007.81), and the nine-year follow-up (2014/618/REKmidt). Written information with illustrations was distributed to the children and their parents, and consent was obtained for all participants (from the parents on behalf of the children).

### Statistics

All data were analyzed for normality with the Kolmogorov-Smirnov test and checked for outliers by Q-Q plots. Skewness and kurtosis were analyzed by g1 and z-score. Characteristics are presented as mean ± standard deviation, median (interquartile range (IQR)), or n [%]. P-values for differences in measures between the 2^nd^ and 3^rd^ trimesters of pregnancy, and differences between measures in girls and boys, were calculated using Student’s *t*-test or Mann Whitney *U* test depending on the distribution of continuous variables. P-values for differences in categorical values were calculated by a Chi-Square test with a *post-hoc* residual analysis, as described by García-pérez et al., when significant (40). To determine the relationships between the intake of RAE and maternal serum retinol and the intake of vitamin D and maternal serum 25(OH)D in the 2^nd^ and in the 3^rd^ trimester, Pearson correlation coefficients (r) were computed.

The main outcomes: child spine BMC and TBS, were normally distributed and used as continuous dependent variables in multivariate linear regression analyses to assess the associations between maternal vitamin A and D serum concentrations in both 2^nd^ and 3^rd^ trimesters of pregnancy and children’s BMC and TBS at nine years of age. Multiple imputation (Regression Method) was used for missing values of serum vitamin A and D in the 3^rd^ trimester. To detect violations of the model assumptions, z-scores of residuals and predictors were plotted. The results from linear regression analyses are presented as mean difference with 95% confidence intervals in spine BMC and TBS per maternal 10 nmol/L 25(OH)D or 0.1 µmol/L retinol, and maternal retinol or vitamin D status categories. Potential confounders were chosen based on assumptions. and were as follows; in children: age, height, sex, vitamin D status, sample season, body weight, birthweight, and gestational age, and in mothers: age, education, smoking, pre-pregnancy BMI, parity, and sample season (for vitamin D only). The potential confounding factors are assumed to influence offspring bone health and are previously described and used in studies with similar exposures, outcomes, and populations (28, 29). The regression models were as follows: crude unadjusted model, model A: adjusted for child age, height and sex, model B: adjusted for A and maternal confounders (age, education, smoking, pre-pregnancy BMI, parity, and sample season (for vitamin D only)), model C: adjusted for A, B and child serum vitamin D status, body weight, birthweight, gestational age and children’s sample season. P-values< 0.05 were considered significant. Additionally, were the models were further adjusted for randomization from the original RCT (30), as well as frequency and intensity of exercise training in pregnancy. A linear regression model using the product of maternal retinol and 25(OH)D serum concentrations as continuous variable was performed, to test a potential interaction of maternal retinol and 25(OH)D status on offspring BMC and TBS. The maternal serum concentration values were centered before they were multiplied together, to reduce multicollinearity. All statistical analyses were performed using SPSS (IBM SPSS, Inc., version 27, 2020) and GraphPad Prism (GraphPad Software, Inc., version 9.2.0, 2021). Figures were made in GraphPad Prism.

## Results

### Characteristics of the mothers during pregnancy

**Figure 1:**
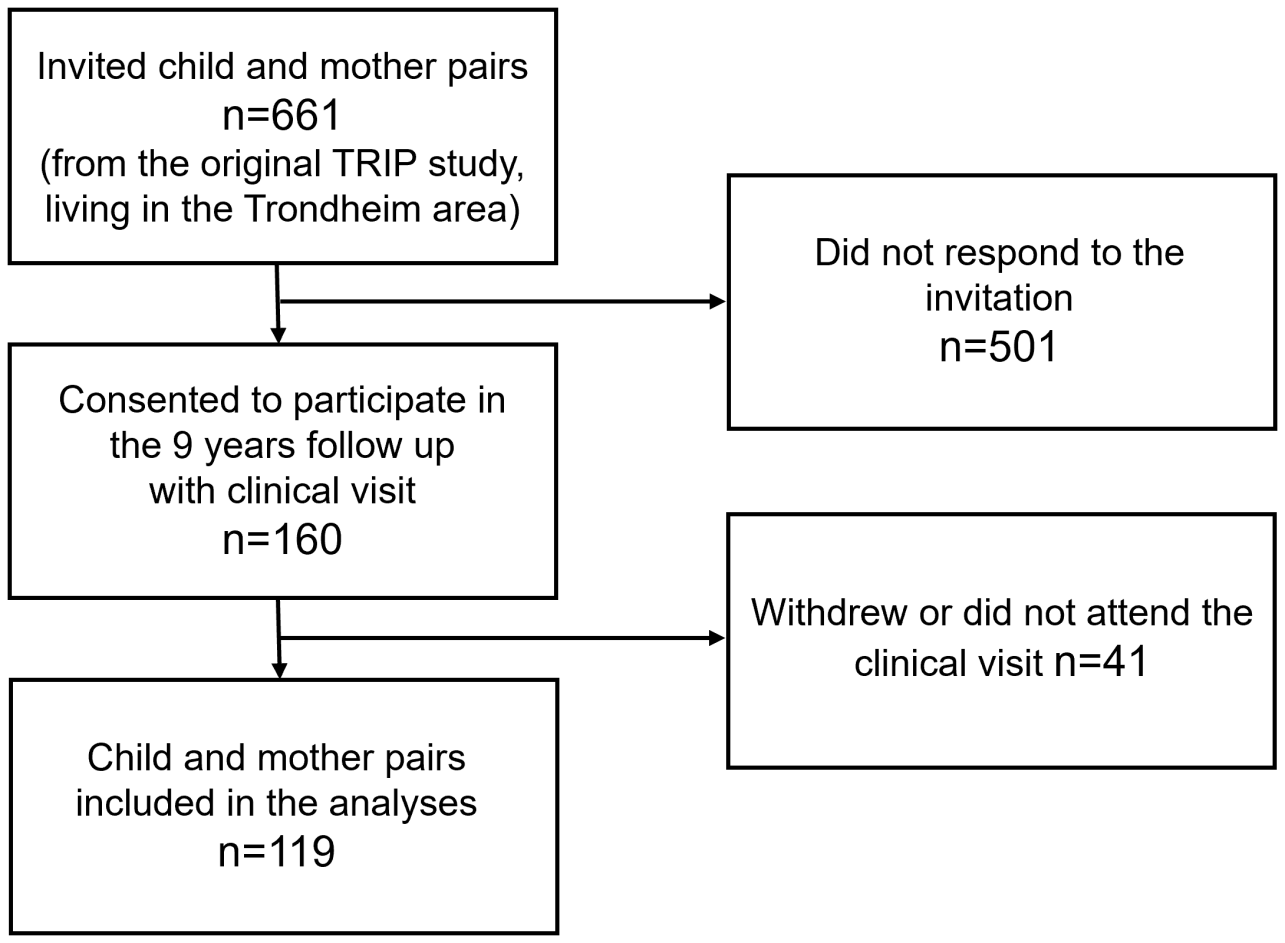
Flow chart of study.

A total of 661 mother and child pairs were invited, and 156 (23.6%) consented to participate in the follow-up study. During the inclusion period, 41 participants withdrew, moved from the region, or did not attend the clinical assessments. Therefore, 119 mother and child pairs were included in the analyses. A flow chart of the study recruitment is presented in **Figure 1**. Maternal and neonatal characteristics (sex distribution, birthweight, and gestational age at birth) among those who participated in the follow-up study and those who did not, are shown in **Table 1**. Except for a higher median age at delivery among women who participated in the follow-up study (31 versus 30 years of age), no differences were observed.

**Table 1:** Mothers’ characteristics during pregnancy.

		Participants in follow-up (n=119)	Did not participate in follow-up (n=542)
Age at inclusion (2^nd^ trimester), years		31 (4)	30 (6)
Age range, years		24-41	19-46
Education	High school ≤ 13 years	10 [8.4]	57 [10.5]
	College/University ≤ 4 years	41 [34.5]	211 [38.9]
	College/University > 4 years	68 [57.1]	274 [50.6]
Married/living with partner		118 [99.2]	528 [97.6]^a^
Parity ≥ 1		57 [47.9]	229 [42.3]
Height, cm		169 ± 6.1	169 ± 6.1
Pre-pregnancy BMI, kg/m^2^		22.7 (3.5)	22.7 (3.7)^b^
BMI at inclusion (2^nd^ trimester), kg/m^2^		24.3 (4.0)	24.4 (3.6)^c^
Smoking during pregnancy		2 [1.7]	3 [0.6]
2^nd^ trimester physical activity			
Days/week with regular exercise training		1 (2)	1 (2)
High intensity exercise training ≥ 3 days/week		15 [12.6]	66 [12.2]
2^nd^ trimester dietary intake* and serum analyses			
Vitamin A, RAE, µg/day		900 (668)	962 (666)
Vitamin D, µg/day		8.9 (9.1)	8.6 (8.3)
Calcium, mg/day		897 (489)	923 (433)
sRetinol, µmol/L		1.4 (0.3)	1.4 (0.3)^a^
0.7-1.1 µmol/L		8 [6.7]	39 [7.2]
> 1.1 µmol/L		111 [93.3]	502 [92.8]
s25(OH)D, nmol/L		65 (34)	63 (32)^a^
< 30 nmol/L		5 [4.2]	28 [5.2]
30-50 nmol/L		28 [23.5]	124 [22.9]
50-75 nmol/L		46 [38.7]	206 [38.1]
> 75 nmol/L		40 [33.6]	183 [33.8]
sPTH, pmol/L		2.7 (1.2)	2.6 (1.2)^a^
sAlbumin, g/L		37 (3.0)	37 (3.0)
sCalcium, mmol/L		2.27 (0.09)	2.27 (0.09)
Sampling season winter and spring (Dec-May)		68 [57.1]	280 [51.7]
Sampling season summer and fall (June-Nov)		63 [42.9]	262 [48.3]
3^rd^ trimester physical activity			
Days/week with regular exercise training		2 (3)	2 (3)
High intensity exercise training ≥ 3 days/week		44 [37.3]^a^	166 [34.2]^d^
3^rd^ trimester dietary intake* and serum analyses			
Vitamin A, RAE, µg/day		899 (630)^c^	865 (608)^d^
Vitamin D, µg/day		9.4 (9.8)^c^	7.9 (8.4)^d^
Calcium, mg/day		921 (521)^c^	947 (448)^d^
sRetinol, µmol/L		1.1 (0.3)^e^	1.1 (0.4)^f^
< 0.7 µmol/L		2 [1.8]	7 [1.5]
0.7-1.1 µmol/L		47 [41.2]	190 [40.7]
> 1.1 µmol/L		65 [57.0]	270 [57.8]
s25(OH)D, nmol/L		60 (43)^e^	59 (37)^f^
< 30 nmol/L		8 [7.1]	36 [7.7]
30-50 nmol/L		29 [25.4]	123 [26.3]
50-75 nmol/L		38 [33.3]	166 [35.5]
> 75 nmol/L		39 [34.2]	142 [30.4]
sPTH, pmol/L		3.4 (2.1)^e^	3.4 (1.7)^f^
sAlbumin, g/L		34 (2.0)^e^	34 (2.0)^f^
sCalcium, mmol/L		2.24 (0.07)^e^	2.25 (0.07)^f^
Sampling season winter and spring (Dec-May)		53 [46.1]^g^	222 [47.5]^f^
Sampling season summer and fall (June-Nov)		62 [53.9]	245 [52.5]
Birth characteristics			
Sex distribution (girls)		62 [52]	260 [48]^a^
Birthweight (g)		3585 (560)	3535 (630)^a^
Gestational age (week) at birth		39.6 ± 1.4	40.0 ± 1.7

Data are in mean ± standard deviation, median (interquartile range) or n [%]. 2^nd^ trimester=gestational week 18-22, 3^rd^ trimester=gestational week 32-36, BMI, body mass index; RAE, retinol activity equivalents; s, serum; PTH, parathyroid hormone. *Intake is calculated from diet and supplements. ^a^missing n=1, ^b^missing n=6, ^c^missing n=2, ^d^missing n=56, ^e^missing n=5, ^f^missing n=75, ^g^missing n=4.

All the included women were of Caucasian origin and the majority had education at college/university level. According to the pre-pregnancy BMI, most (76.5%) had a BMI within the normal range (18.5-24.9 kg/m^2^), three women (2.5%) were underweight (BMI< 18.5 km/m^2^), and 21 (17.6%) and four (3.4%) were overweight or obese (BMI > 25 and BMI > 30 kg/m^2^, respectively). The median vitamin A (RAE) and calcium intake in both trimesters were above the current Norwegian and EFSA recommendations (> 700 µg/day and > 900 mg/day) (37, 41, 42). In comparison, the U.S. Department of Health and Human Services (U.S. HHS) Dietary Guidelines for Americans recommend a higher RAE and calcium intake during pregnancy (> 770 µg/day and > 1000 mg/day, respectively) (43), than the Norwegian and EFSA guidelines. A daily RAE intake below 700 µg/day was reported by 16 (13.4%) and 21 (17.9%) participants in the 2^nd^ and 3^rd^ trimesters, respectively. A daily vitamin D intake over 10 µg/day, which is recommended by the Norwegian Directorate of Health (42), was reported by less than half of the women (41.2% and 44.4% in the 2^nd^ and 3^rd^ trimesters, respectively). Approximately 25% of the women reported a vitamin D intake above 15 µg/day during pregnancy, which is the recommendation by EFSA and the U.S. HSS (21, 43).

### Serum analyses in mothers

As shown in **Table 1**, none of the women had vitamin A deficiency (sRetinol< 0.7 µmol/L) in the 2^nd^ trimester, and only two (1.8%) in the 3^rd^ trimester. Vitamin A insufficiency (sRetinol 0.7-1.1 µmol/L) was observed in eight women (6.7%) in the 2^nd^ trimester, and in 47 women (41.2%) in the 3^rd^ trimester. Altogether, 33 (27.7%) had vitamin D deficiency (s25(OH)D< 50 nmol/L) in the 2^nd^ trimester, versus 37 (32.5%) in the 3^rd^ trimester. Five (4.2%) and eight (7.1%) women had extreme vitamin D deficiency (s25(OH)D< 30 nmol/L) in the 2^nd^ and 3^rd^ trimesters, respectively.

**Table 2** shows maternal serum concentrations of components of the vitamin D endocrine system in the two trimesters of pregnancy. A significant decrease occurred in serum 25(OH)D, albumin, total calcium, and free and bioavailable vitamin D from the 2^nd^ to the 3^rd^ trimester, whereas PTH and vitamin D binding protein increased. A significant rise in 1,25(OH)_2_D concentrations from the 2^nd^ to the 3^rd^ trimester was also observed in the subgroup of participants with available 1,25(OH)_2_D measurements (n=53).

**Table 2:** Differences in components of the vitamin D endocrine system in serum between the 2^nd^ and the 3^rd^ trimester of pregnancy.

n=114^a^	2^nd^ trimester	3^rd^ trimester	MD from 2^nd^ to 3^rd^	p-value 2^nd^ vs 3^rd^
25(OH)D, nmol/L	65 (34)	60 (43)^a^	-2.0	**< 0.001**
PTH, pmol/L	2.7 (1.2)	3.4 (2.1)	0.8	**< 0.001**
Albumin, g/L	37 (3.0)	34 (2.0)	-3.0	**< 0.001**
Calcium, mmol/L	2.27 (0.09)	2.24 (0.07)	-0.03	**< 0.001**
1,25(OH)_2_D, pmol/L^b^	195 (61.5)	217 (82.5)	22	**< 0.001**
DBP, µmol/L	5.70 (1.20)	6.20 (1.13)	0.60	**< 0.001**
Calculated free vitamin D, pmol/L	15.3 (7.10)	13.0 (8.66)	-1.53	**< 0.001**
Albumin bound vitamin D, nmol/L	4.87 (2.37)	4.08 (2.71)	-0.91	**< 0.001**
Bioavailable vitamin D, nmol/L	4.88 (2.37)	4.10 (2.72)	-0.91	**< 0.001**
Free vitamin D, %	7.75 (1.44)	6.60 (1.00)	-1.07	**< 0.001**

Data are in median (interquartile range) and median difference (MD). ^a^Calculations based on n=114, as n=5 missing in the 3^rd^ trimester, ^b^calculations based on subgroup with n=53. PTH, parathyroid hormone; DBP, vitamin D binding protein. Numbers in bold indicates significant p-values (p < 0.05).

### Correlations of nutrient intake and vitamin status in mothers

There were no significant correlations between RAE intake (µg/day) and maternal serum retinol (µmol/L) in the 2^nd^ and the 3^rd^ trimester of pregnancy (r=0.02, n=119, p=0.857 and r=0.08, n=117, p=0.416, respectively). Maternal vitamin D intake (µg/day) showed a weak positive correlation with serum 25(OH)D (mmol/L) in the 2^nd^ trimester (r=0.30, n=119, p=0.002), and a weak positive correlation with serum 25(OH)D (mmol/L) in the 3^rd^ trimesters (r=0.27, n=117, p=0.003).

### Characteristics of the children

Childrens’ characteristics at birth and at nine years of age are presented in **Table 3**. A total of 119 children (62 girls and 57 boys) were included in the analyses. The neonatal characteristics differed between the sexes, as boys had a higher median birthweight than girls (3735 g versus 3377 g, respectively), and a higher proportion of the boys had a birthweight over 4000 g. The number of children with low birthweight (< 2500 g) was similar between girls and boys (1.6% and 3.5%), and the mode of delivery (cesarean and vaginal) did not differ between the sexes. All children were term-born (i.e. gestational week 37-42), except one girl (gestational week 36) and one boy (gestational week 34). According to iso-BMI categories (34), almost 90% of the children had normal body weight, 5% were underweight, and ~6% were overweight/obese. The distribution of age, height, weight, BMI, and iso-BMI categories did not differ between the sexes.

**Table 3:** Children’s characteristics at birth and at nine years follow-up.

	Total n=119	Girls n=62 [52%]	Boys n=57 [48%]	p-value Girls vs Boys
At birth				
Birthweight, g	3585 (560)	3377 (450)	3735 (555)	**< 0.001**
Birthweight group				**0.001** ^#^
< 2500 g	3 [2.5]	1 [1.6]	2 [3.5]	0.510^##^
2500-4000 g	94 [79.0]	57 [91.9]	37 [64.9]	**< 0.001^##^**
> 4000 g	22 [18.5]	4 [6.5]	18 [31.6]	**< 0.001** ^##^
Gestational week at birth	39.6 ± 1.41	39.3 ± 1.34	40.0 ± 1.41	**0.007**
Mode of delivery^a^				0.515^#^
Cesarean delivery	13 [11.3]	8 [13.1]	5 [9.3]	
Vaginal birth	102 [88.7]	53 [86.9]	49 [90.7]	
At nine years follow-up				
Age, years	9.04 ± 0.42	9.07 ± 0.38	9.01 ± 0.47	0.503
Age, range, years	8.08-10.0	8.25-10.0	8.08-9.92	
Weight, kg	31.0 (5.0)	30 (3.3)	32 (6.5)	0.108
Height, cm	137 (9.00)	136 (6.25)	138 (10.5)	0.155
BMI, kg/m^2^	16.4 (1.8)	16.4 (1.7)	16.5 (1.6)	0.232
isoBMI category				0.727^#^
Underweight	6 [5.0]	4 [6.5]	2 [3.5]	
Normal	106 [89.1]	54 [87.0]	50 [91.2]	
Overweight/Obese	7 [5.9]	4 [6.5]	3 [5.3]	
DXA measurements				
Spine BMC, g	22.7 ± 3.76	22.2 ± 3.39	23.4 ± 4.06	0.090
Spine BMD, g/cm^2^	0.593 ± 0.069	0.597 ± 0.069	0.590 ± 0.068	0.571
Spine TBS	1.294 ± 0.082	1.260 ± 0.077	1.331 ± 0.071	**< 0.001**
Whole body BMC^b^, g	1123 (238)	1073 (197)	1170 (235)	**0.003**
Whole body BMD^b^, g/cm^2^	0.826 ± 0.071	0.804 ± 0.070	0.851 ± 0.064	**< 0.001**
Femur BMC^c^, g	16.6 (21.4)	17.6 (23.5)	16.0 (18.9)	0.937
Femur BMD^c^, g/cm^2^	0.776 ± 0.073	0.761 ± 0.076	0.793 ± 0.067	**0.023**
Total hip BMC^d^, g	15.3 (4.15)	14.9 (3.91)	16.7 (4.65)	**0.009**
Total hip BMD^d^, g/cm^2^	0.694 (0.114)	0.666 (0.123)	0.737 (0.122)	**< 0.001**
Biochemical analyses				
sAlbumin^e^, g/L	45 (3.0)	45 (2.0)	43 (2.0)	**< 0.001**
sCalcium^e^, mmol/L	2.48 ± 0.07	2.51 ± 0.07	2.46 ± 0.06	**< 0.001**
sPTH^e^, pmol/L	2.3 (1.5)	2.3 (1.4)	2.1 (1.7)	0.864
s25(OH)D^e^, nmol/L	59.2 ± 16.1	59.5 ± 15.5	58.9 ± 16.8	0.851
s25(OH)D^e^ category				0.607^#^
< 30 nmol/L	4 [3.5]	1 [1.6]	3 [5.5]	
30-50 nmol/L	27 [23.3]	16 [26.3]	11 [20.0]	
50-75 nmol/L	65 [56.0]	33 [54.1]	32 [58.2]	
> 75 nmol/L	20 [17.2]	11 [18.0]	9 [16.3]	
Sampling season^f^				0.181^#^
winter and spring (Dec-May)	75 [64.7]	36 [59.0]	39 [70.9]	
summer and fall (June-Nov)	41 [34.5]	25 [41.0]	16 [29.1]	

Data in mean ± standard deviation, median (IQR) or n [%], p-values by Student’s t-test or Mann Whitney U test, ^#^overall p-value by Chi-Square test, ^##^p-value by posthoc residual analyses. BMI, body mass index; DXA, dual X-ray absorptiometry; BMC, bone mineral content; BMD, bone mineral density; TBS, trabecular bone score; s, serum; PTH, parathyroid hormone. ^a^missing n=4 (1G, 3B), ^b^missing n=10 (6G, 4B), ^c^missing n=10 (4G, 6B), ^d^missing n=21 (9G, 12B), ^e^missing n=4 (2G, 2B), ^f^missing n=3 (1G, 2B). Numbers in bold indicates significant p-values (p < 0.05).

### DXA measurements and serum analyses in the children

As shown in **Table 3**, the mean value for the main outcome, spine BMC, was 22.7 ± 3.76 g, and did not differ between the sexes, while spine TBS was significantly higher in boys than in girls (1.331 ± 0.071 versus 1.260 ± 0.077, respectively, mean difference: -0.072, 95% CI: -0.100 to -0.044, p< 0.001). The boys also had higher whole body and total hip BMC and BMD, and distal femur BMD, than the girls.

The mean serum 25(OH)D in the children was 59.2 ± 16.1 nmol/L and did not differ between girls and boys. A total of 31 children (26.8%) were vitamin D deficient (s25(OH)D< 50 nmol/L). Four children (3.5%) were extremely vitamin D deficient (s25(OH)D< 30 nmol/L). The girls had significantly higher serum levels of total calcium and albumin than the boys, however, all children had serum levels of albumin and calcium within the reference range for children of their age (44, 45). There was no difference in serum PTH levels between sexes, and PTH concentrations ranged from 0.4 to 4.7 pmol/L in girls and from 0.6 to 6.2 pmol/L in boys.

### Associations with vitamin A

**Figure 2:**
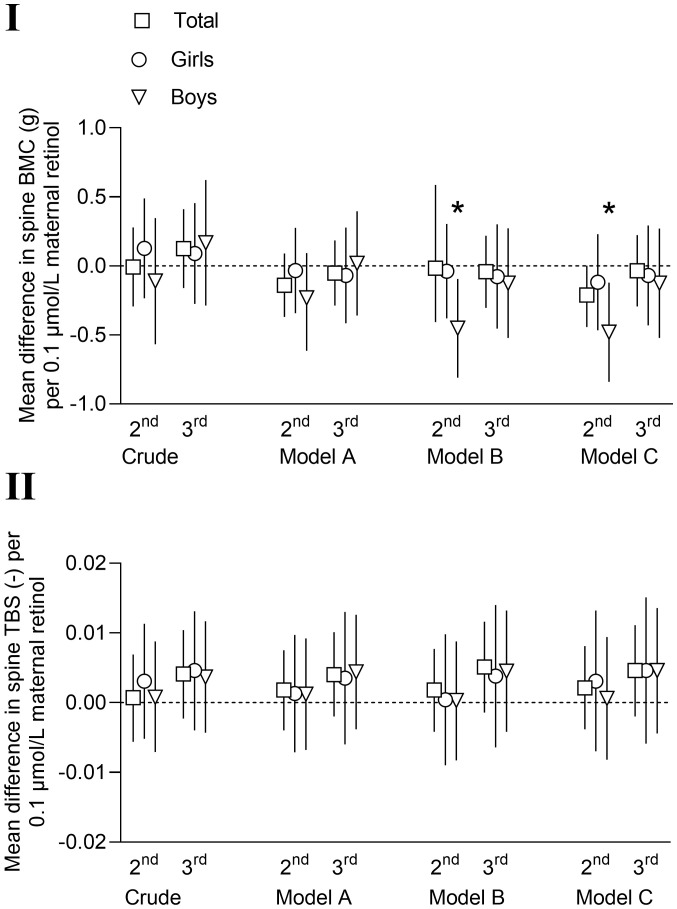
Mean differences with 95% confidence intervals in offspring spine bone mineral content (BMC, g) (I) and spine trabecular bone score (TBS) (II) at nine years of age per 0.1 µmol/L maternal serum retinol in 2^nd^ and 3^rd^ trimester. Model A: adjusted for child age and height (and sex in total column). Model B: model A + maternal age, parity, education, smoking during pregnancy and pre-pregnancy body mass index (kg/m^2^). Model C: models A + B + child serum vitamin 25(OH)D, child body weight, birthweight, gestational age at birth and child serum sample season. *=p< 0.05.

Regression models showed that maternal serum retinol concentrations as a continuous variable in the 2^nd^ and 3^rd^ trimester of pregnancy, were not associated with spine BMC or TBS at nine years in the children in either the crude or any of the multivariable models (**Figure 2**; **Supplementary Table 1**). However, when stratified for sex, a significant negative association was observed between retinol in the 2^nd^ trimester and spine BMC in the boys in adjusted models B and C (**Figure 2**; **Supplementary Table 1**). Mean spine BMC among offspring from mothers with insufficient or deficient serum levels of retinol in the 2^nd^ and 3^rd^ trimester were similar to the values among offspring of mothers with sufficient levels, also when adjusted for confounders (**Table 4**), and when stratified for sex and the randomization from the original study (30) and including frequency and intensity of regular exercise training as confounders (data not shown).

**Table 4:** Mean difference with 95% confidence interval (CI) in offspring spine bone mineral content (BMC, g) at nine years of age with maternal vitamin A (serum retinol) ≥ 1.1 µmol/L as reference category, in 2^nd^ and 3^rd^ trimester of pregnancy.

Maternal sRetinol	n [%]	β and 95% CI in BMC (g)			
		Crude	Model A	Model B	Model C
2^nd^ trimester	119 [100]				
≥ 1.1 µmol/L	111 [93.3]	Reference	Reference	Reference	Reference
< 1.1 µmol/L	8 [6.7]	-0.7980 (-3.532 – 1.937)	0.7092 (-1.4236 – 2.842)	1.064 (-1.152 – 3.280)	1.012 (-1.190 – 3.214)
3^rd^ trimester^a^	119 [100]				
≥ 1.1 µmol/L	70 [58.8]	Reference	Reference	Reference	Reference
< 1.1 µmol/L	49 [41.2]	-0-6891 (-2.077 – 0.6985)	0.0880 (-0.9963 – 1.172)	0.1126 (-0.9182 – 1.143)	0.3258 (-1.008 – 1.073)

Model A: adjusted for child sex, age and height. Model B: model A + maternal age, parity, education, smoking during pregnancy and pre-pregnancy body mass index (kg/m^2^). Model C: models A + B + child serum vitamin 25(OH)D, child body weight, birthweight, gestational age at birth and child serum sample season. ^a^n=5 missing values were imputated.

### Associations with vitamin D

**Figure 3:**
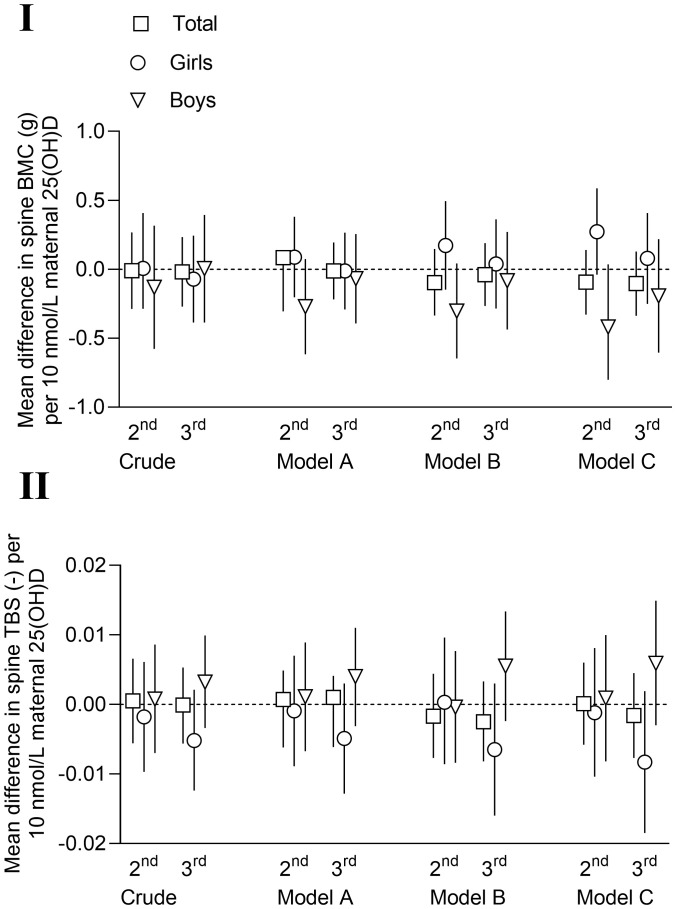
Mean differences with 95% confidence intervals in offspring spine bone mineral content (BMC, g) (I) and spine trabecular bone score (TBS) (II) at nine years of age per 10 nmol/L maternal serum vitamin D (25(OH)D) in 2^nd^ and 3^rd^ trimester. Model A: adjusted for child age and height (and sex in total column). Model B: model A + maternal age, parity, education, smoking during pregnancy, pre-pregnancy body mass index (kg/m^2^) and sample season. Model C: models A + B + child serum vitamin 25(OH)D, child body weight, birthweight, gestational age at birth and child serum sample season.

Regression models showed no associations between maternal serum 25(OH)D concentrations as a continuous variable in the 2^nd^ and 3^rd^ trimesters of pregnancy and offspring spine BMC or TBS at nine years of age (**Figure 3**; **Supplementary Table 2**). These null associations were similar in all multivariable models, also when stratified for sex (**Figure 3**; **Supplementary Table 2**). Mean spine BMC in offspring of mothers with vitamin D deficiency (s25(OH)D< 50 nmol/L) in the 2^nd^ and 3^rd^ trimester were similar to the values for offspring of mothers with s25(OH)D levels above 50 nmol/L, also when adjusted for confounders (**Table 5**). Sex stratification, stratification for the randomization in the original study (30) and including frequency and intensity of regular exercise training as confounders, did not affect these results (data not shown).

**Table 5:** Mean difference with 95% confidence interval (CI) in offspring spine bone mineral content (BMC, g) at nine years of age with maternal vitamin D (serum 25(OH)D) ≥ 50 nmol/L as reference category, in 2^nd^ and 3^rd^ trimester of pregnancy.

Maternal s25(OH)D	n [%]	β and 95% CI in BMC (g)			
		Crude	Model A	Model B	Model C
2^nd^ trimester	119 [100]				
≥ 50 nmol/L	86 [72.3]	Reference	Reference	Reference	Reference
< 50 nmol/L	33 [27.7]	0.5163 (-1.0126 – 2.045)	0.5696 (-0.6506 – 1.790)	0.3587 (-0.9100 – 1.327)	0.1055 (-1.156 – 1.367)
3^rd^ trimester^a^	119 [100]				
≥ 50 nmol/L	80 [67.2]	Reference	Reference	Reference	Reference
< 50 nmol/L	39 [32.8]	-0.5360 (-2.004 – 0.9345)	-0.6871 (-1.864 – 0.4900)	-0.7690 (-2.012 – 0.4744)	-0.5150 (-1.721 – 0.6912)

Model A: adjusted for child sex, age and height. Model B: model A + maternal age, parity, education, smoking during pregnancy, pre-pregnancy body mass index (kg/m^2^) and sample season. Model C: models A+ B + child serum vitamin 25(OH)D, child body weight, birthweight, gestational age at birth and child serum sample season. ^a^n=5 missing values were imputated.

### Associations with the synergy of vitamin A and vitamin D

No associations were seen between the product of centered values of maternal serum retinol and 25(OH)D as interaction (moderator) effect in the 2^nd^ and 3^rd^ trimesters of pregnancy and offspring spine BMC or TBS at nine years of age. 2^nd^ trimester; BMC: F(1,117)=1.54, p=0.218, R^2 ^= 0.013 and TBS: F(1,117)=0.53, p=0.470, R^2 ^= 0.004. 3^rd^ trimester; BMC: F(1,117)=0.109, p=0.741, R^2 ^= 0.001 and TBS: F(1,117)=0.066, p=0.798, R^2 ^= 0.001.

## Discussion

In the current study we assessed potential associations between maternal vitamin A (serum retinol) and D (serum 25(OH)D) in the 2^nd^ and 3^rd^ trimester of pregnancy and bone parameters in offspring at the age of nine years. We observed a negative association between serum levels of vitamin A in the 2^nd^ trimester and spine BMC in boys, when adjusted for maternal and child confounders. No other associations were found between maternal vitamin A and D status and offspring spine BMC and TBS. We observed a high prevalence of vitamin A insufficiency and vitamin D deficiency in the 3^rd^ trimester, 43.0% and 32.5%, respectively. Moreover, a significant rise in maternal 1,25(OH)_2_D and PTH serum concentrations occurred from the 2^nd^ to the 3^rd^ trimester.

The finding of a negative association in two of the adjusted regression models between maternal serum retinol in the 2^nd^ trimester and spine BMC in boys, is in contrast to our previous study, where we observed a positive association between the average serum vitamin A (retinol) in gestational weeks 17, 33 and 37 and offspring peak bone mass (spine BMD) at the age of 26 years, when adjusted for confounders (22). A limitation of the previous study was the small sample size (n=41 mother and offspring pairs). A discrepancy between the two studies is that the mean maternal levels of serum retinol was higher in the previous study (1.66 µmol/L versus 1.25 µmol/L) and with a larger heterogeneity in retinol concentrations, which may have influenced the results. The difference in serum retinol levels may be attributed to alteration in the dietary intake of vitamin A in the two time periods the studies were conducted (1986–88 versus 2007–09).

A negative association between maternal serum retinol in late pregnancy and neonatal whole-body BMC, but a positive association for serum β-carotene, was found in a previous study from the UK by Handel et al. (23). This study did not stratify for sex, but adjusted for sex, age, and gestational age at birth in the models (23). Tobias et al. investigated the relation between a wide range of maternal nutritional factors, assessed by a FFQ, in the 3^rd^ trimester, and child bone mass at nine years of age, and found no significant association to either vitamin D, retinol, or β-carotene dietary intake (46). However, the inaccuracies of FFQs in assessing intake and estimating vitamin status should be considered. Interestingly, higher concentrations of retinol are reported in umbilical cord blood than in the pregnant women themselves, indicating an acceleration in the intrauterine transfer rate of retinol in cases of maternal vitamin A deficiency or insufficiency (47).

The null association between maternal vitamin D and offspring bone outcomes in the present study complies with our previous report, where we found no associations between the mean level of serum 25(OH)D in gestational weeks 17, 33 and 37, and peak bone mass and trabecular bone score in offspring at 26 years of age (24). Also, no associations between maternal vitamin D status and offspring BMC were found in two large European studies with n=4815 six years old children (28), and n=3690 10 years old children (29).

However, associations between maternal vitamin D and offspring BMC have also been reported. Javaid et al. described lower whole body and spine BMC in 198 nine year old offspring of women with serum 25(OH)D< 27.5 nmol/L, compared to women with vitamin D sufficiency (s25(OH)D > 50 nmol/L) in the 3^rd^ trimester (26). Moreover, Zhu et al. reported lower peak bone mass (whole body BMC and BMD) in offspring (n=341) at 20 years of age of mothers with 25(OH)D< 50 nmol/L in mid-pregnancy (27). Hyde et al. found a positive association between circulating 25(OH)D in early pregnancy and spine BMC and BMD in boys (n=93), but not girls (n=88), at 11 years of age (25). Of these studies, the two latter are most comparable to the current, as they measured bone content and density at the same sites at approximately the same offspring age. The Javaid et al. study, on the other hand, described a linear dose-response association between low maternal 25(OH)D concentrations in the 3^rd^ trimester and low whole body and spine BMC in the children at nine years of age (26). It must be noted that the percentage of women with vitamin D deficiency (defined in Javaid et al. study as 25(OH)D< 27.5 nmol/L), was almost six times higher than in the current study (18% versus 3.4%). It might be that offspring bone parameters are mainly affected when mothers are highly vitamin D deficient during pregnancy. In support of this, a study from Denmark reported that vitamin D supplementation of 2800 IU/day versus 400 IU/day from mid-pregnancy, resulted in higher whole body BMC and BMD in the offspring at 6 years of age, with the most pronounced effects among children of mothers with insufficient serum 25(OH)D levels or born during winter/spring (48). Also, the MAVIDOS trial *post-hoc* analyses suggested that supplementation to mothers with serum 25(OH)D< 30 nmol/L is beneficial for offspring bone measures in infancy (49).

In line with previous studies (8, 31), we observed a rise in serum levels of the active form of vitamin D, 1,25(OH)_2_D, during pregnancy, whereas 25(OH)D decreased. Free and bioavailable vitamin D also declined during pregnancy, while PTH and vitamin D binding protein increased along with 1,25(OH)_2_D. The increase in 1,25(OH)_2_D in pregnancy has been suggested as a compensatory mechanism to uphold serum 1,25(OH)_2_D levels in a state of maternal vitamin D insufficiency (24). However, recent studies states that mechanisms independent of maternal 25(OH)D status secure the fetus’ access to sufficient calcium necessary for mineral metabolism and skeletal development (9). These mechanisms are regulated by PTH-related protein (PRHrP), and to a lesser extent by PTH (50). On the other hand, it must be underlined that significant amounts of vitamin D is highly important during pregnancy to protect the mother as well as the fetus (51). According to Czech-Kowalska et al. is maternal 25(OH)D status the key determinant for umbilical cord blood 25(OH)D concentrations (52). This implies that high levels of maternal circulating vitamin D are of importance to secure that the offspring begins life with adequate vitamin D stores necessary for further skeletal development (11). Hollis et al. suggest to strive for a circulating 25(OH)D concentration of at least 100 nmol/L during pregnancy to optimize the conversion of 25(OH)D to 1,25(OH)_2_D, and thereby reduce the risk of comorbidities of pregnancy and better outcomes for both mother and child (51). Cord blood concentration of 25(OH)D is previously reported to be 50-70% of the maternal levels (53, 54).

Adequate intake of calcium and vitamin D in childhood is essential for accrual of mineral in the skeleton. Unfortunately, we do not have data on calcium and vitamin D intake in the children, but the children’s calcium, PTH and 25(OH)D serum levels were analyzed, and the latter was adjusted for in the regression models. The adjustment did not affect the results, but it is likely an important factor to account for as the child’s vitamin D status, in addition to gender and age, is an important determinant of bone mass in life (55). The children’s PTH serum levels were not adjusted for in the regression models, as the reference levels for circulating PTH in children are not fully established, and varying cut-offs are reported (56, 57).

In the present study, 6.7% (2^nd^ trimester) and 43% (3^rd^ trimester) of the participants had suboptimal serum retinol concentrations, and 13.4% and 17.9% in the 2^nd^ and 3^rd^ trimesters, respectively, reported a lower intake of RAE than the recommended 700 µg/day (37, 41, 42). A total of 16.0% and 23.1% of the women in the 2^nd^ and 3^rd^ trimesters, respectively, reported a daily intake below the recommendations from the American College of Obstrecians and Gynecologists (ACOG) and the U.S. HSS of 770 µg/day vitamin A in pregnancy (43, 58). However, the reported RAE intake and serum retinol concentrations in pregnancy did not correlate in our study, supporting the suggestion that retinol in circulation only reflect the liver storage of vitamin A in extreme cases, and thus might not correlate with intake or clinical signs of deficiency (59).

We found that 27.7% and 32.5% of the women were vitamin D deficient (s25(OH)D< 50 nmol/L) in the 2^nd^ and 3^rd^ trimesters, respectively, despite that the study population consisted of well-educated women with low-risk pregnancies. These data correspond with our previous study of the total population of 855 women from the original TRIP study (31), and support the worldwide observations of a high prevalence of VDD in pregnancy. Norway is situated at northern latitudes and UVB-mediated dermal synthesis of vitamin D is absent in the dark seasons. Vitamin D must therefore be obtained through diet and supplements. We found that reported vitamin D intake in the mothers were positively correlated to the maternal serum 25(OH)D concentrations in both trimesters. Like in most countries, the diet in Norway has a low content of vitamin D, and vitamin D-fortified foods are sparse (31, 60). It is of concern that less than half of the women adhered to the recommendations by the Norwegian Directorate of Health, of a minimum 10 µg/day intake of vitamin D (42). The lack of adherence is in line with results from the large Norwegian Mother and Child Cohort, which included nutrition data from over 60 000 women during pregnancy, of which less than 40% of the participants reported a daily vitamin D intake above 10 µg (61).

Vitamin A and D are suggested to exert antagonistic effects, and vitamin A may inhibit vitamin D maintenance of the calcium balance (62). In the large Women’s Health Initiative (WHI) prospective study including over 75 000 women, high retinol intake is associated with a modest increased fracture risk only in women with low vitamin D intake (63). Rajwar et al. recently published an overview of systematic reviews on the effects of vitamin A, vitamin D and calcium fortification and supplementation on the nutritional status of women in reproductive age (64). In this overview, they concluded that supplementation of vitamin A increased maternal serum and breast milk retinol concentrations, and reduced the risk of anemia and maternal clinical infection (64). However, in the current study, maternal serum retinol and 25(OH)D in combination were not associated with offspring BMC or TBS.

The sexually dimorphic effect that we found concerning vitamin A has not been observed previously and must be interpreted with caution and within the constraints of our limitations. Hyde et al. reported positive associations between maternal vitamin D in early pregnancy and spine BMC in boys, but not girls, at 11 years of age (25). Also, Percival et al. who included 400 mother and child pairs, found that higher maternal 25(OH)D concentration in the 2^nd^ trimester was associated with a lower fracture risk in boys, whereas higher maternal 25(OH)D concentration in the 3^rd^ trimester was associated with an increased fracture risk in girls (65). Sex differences in associations between maternal factors and BMC in offspring may be explained by sexual dimorphism of the placenta or pubertal stage differences, and these differences may no longer be evident in adulthood.

### Strengths and limitations

The current study has several strengths. Firstly, we have analyzed serum retinol and 25(OH)D in two trimesters of pregnancy, 1,25(OH)_2_D in a subgroup of the women, and the children’s serum levels 25(OH)D. Secondly, DXA measurements of the children were performed at recommended clinically relevant sites and we included spine BMC and TBS in the analyzes. Finally, we had thorough information about the participants, i.e. socioeconomic factors and sample season, which we adjusted for in the statistical models.

The main limitation of this study is the relatively low sample size (n=119 mother and child pairs). A low percentage of women from the initial RCT living in Trondheim, accepted the invitation and participated in this follow-up study (18%). There may also have been some selection bias in the initial study, where the pregnant women who accepted participation in the RCT might be healthier than the average population due to the exercise component of the intervention.

Another limitation is the use of serum retinol as a marker for vitamin A status. Serum retinol can be useful for assessing VAD, however, assessment of borderline sufficient vitamin A status might be affected, since retinol is one of several vitamin A forms (66). The vitamin D analyses in mothers and children were performed at two different timepoints and unfortunately by two different methods, due to available methodology at the Hospital laboratory. Maternal serum 25(OH)D analyses were performed in 2016 by an ECLIA and serum from children were analyzed for 25(OH)D in 2021 with a LC-MS/MS method, of which the latter is considered the gold standard. Also, there were few women with vitamin A deficiency in our study population, and even though vitamin D deficiency was prevalent, extreme vitamin D deficiency was observed in few of the women compared to other studies. We did not have access to cord blood for measurements of retinol and 25(OH)D, so we could not establish the level of retinol and 25(OH)D exposure to the neonate. Data on 1,25(OH)_2_D was available for less than half of the women.

## Conclusions

Maternal serum retinol in the 2^nd^ trimester was negatively associated with spine BMC and TBS in nine years old boys, but not girls, when adjusted for maternal and child confounders. No associations between maternal 25(OH)D in the 2^nd^ and 3^rd^ trimesters and spine BMC and TBS in the children were seen in neither the crude nor the adjusted regression models. There were no interaction effects of maternal retinol and 25(OH)D on offspring bone parameters in the 2^nd^ or the 3^rd^ trimesters. We observed a higher prevalence of vitamin A insufficiency and vitamin D deficiency in pregnancy than anticipated among these well-educated women. An increase in the active form of vitamin D (1,25(OH)_2_D) and PTH during pregnancy were observed across different concentrations of 25(OH)D. The implications of optimal vitamin status and need for vitamin supplementation in pregnancy to improve offspring bone health, remains a subject for further investigations.

## Data availability statement

The original contributions presented in the study are included in the article/**Supplementary Material**. Further inquiries can be directed to the corresponding author.

## Ethics statement

The studies involving humans were approved by Norwegian Regional Committees for Medical and Health Research (REK) was given for the original TRIP-study (4.2007.81), and the nine-year follow-up (2014/618/REKmidt). The studies were conducted in accordance with the local legislation and institutional requirements. Written informed consent for participation in this study was provided by the participants’ legal guardians/next of kin. Written informed consent was obtained from the individual(s), and minor(s)’ legal guardian/next of kin, for the publication of any potentially identifiable images or data included in this article.

## Author contributions

AS: Conceptualization, Formal analysis, Project administration, Writing – original draft, Writing – review & editing, Methodology, Visualization. MM: Data curation, Methodology, Writing – review & editing. MG: Data curation, Writing – review & editing. TB: Data curation, Methodology, Writing – review & editing. PT: Methodology, Writing – review & editing. SS: Conceptualization, Funding acquisition, Project administration, Writing – review & editing. US: Conceptualization, Funding acquisition, Project administration, Writing – review & editing.
