# Supplementary material for: Maternal vitamin A and D status in second and third trimester of pregnancy and bone mineral content in offspring at nine years of age
Source: Front Endocrinol (Lausanne). 2024 Jun 28;15:1417656. doi: 10.3389/fendo.2024.1417656 (PMC11239386; doi:10.3389/fendo.2024.1417656)
Supplement: Supplementary file 1 [file Table_1.docx]

Supplementary Material 1

# Maternal vitamin A and D in second and third trimester of pregnancy and offspring bone measures at nine years of age

Stunes, Astrid Kamilla*^1,2^, Mosti, Mats Peder^1,3^, Børsting, Torunn^2,4^, Thorsby, Per Medbøe^5^, Stafne, Signe Nilssen^4,6^, Syversen, Unni^1,7^.

^1^Clinical and Molecular Medicine, Faculty of Medicine and Health Sciences, Norwegian University of Science and Technology, Trondheim (NTNU), Norway

^2^Center for Oral Health Services and Research, Mid-Norway (TkMidt), Trondheim, Norway

^3^Department of Research and Development, Clinic of Substance Use and Addiction Medicine, St. Olavs University Hospital, Trondheim, Norway

^4^Department of Public Health and Nursing, Norwegian University of Science and Technology (NTNU), Trondheim, Norway

^5^Hormone Laboratory, Department of Medical Biochemistry, Oslo University Hospital, Aker, Oslo, Norway

^6^Clinic of Rehabilitation, St. Olavs Hospital, Trondheim University Hospital, Norway

^7^Department of Endocrinology, Clinic of Medicine, St. Olavs University Hospital, Trondheim, Norway

*** Correspondence:** Astrid Kamilla Stunes, kamilla.stunes@ntnu.no

**
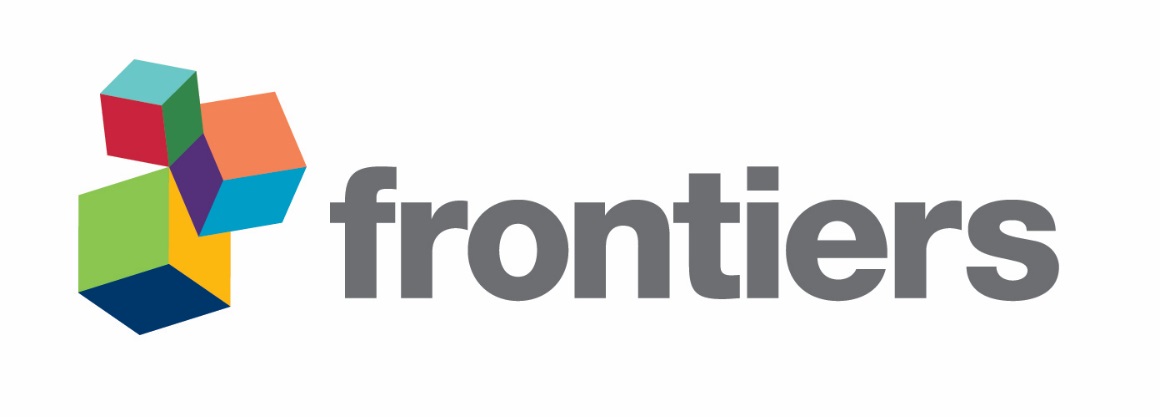
**

|  | **Total, n=119** | | **Girls, n=62 [52%]** | | **Boys, n=57 [48%]** | |
| --- | --- | --- | --- | --- | --- | --- |
| **Crude** | **β** | **95% CI** | **β** | **95% CI** | **β** | **95% CI** |
| 2^nd^ trimester |  |  |  |  |  |  |
| Spine BMC, g | -0.0078 | -0.2942 – 0.2787 | 0.1261 | -0.2357 – 0.4880 | -0.1100 | -0.5673 – 0.3473 |
| Spine BMD, g/cm^2^ | 0.0048 | -0.0003 – 0.0099 | 0.0064 | -0.0008 – 0.0136 | 0.0029 | -0.0048 – 0.0105 |
| Spine TBS, - | 0.0007 | -0.0056 – 0.0069 | 0.0031 | -0.0052 – 0.0113 | 0.0008 | -0.0071 – 0.0088 |
| 3^rd^ trimester |  |  |  |  |  |  |
| Spine BMC, g | 0.1246 | -0.1610 – 0.4103 | 0.0897 | -0.2751 – 0.4546 | 0.1673 | -0.2881 – 0.6227 |
| Spine BMD, g/cm^2^ | 0.0028 | -0.0025 – 0.0081 | 0.0030 | -0.0045 – 0.0105 | 0.0026 | -0.0050 – 0.0102 |
| TBS, - | 0.0041 | -0.0023 – 0.0104 | 0.0046 | -0.0040 – 0.0131 | 0.0037 | -0.0043 – 0.0117 |
| **Model A** |  |  |  |  |  |  |
| 2^nd^ trimester |  |  |  |  |  |  |
| Spine BMC, g | -0.1404 | -0.3708 – 0.0899 | -0.0336 | -0.3426 – 0.2754 | -0.2311 | -0.6148 – 0.0926 |
| Spine BMD, g/cm^2^ | 0.0029 | -0.0019 – 0.0078 | 0.0045 | -0.0024 – 0.0114 | 0.0012 | -0.0058 – 0.0082 |
| Spine TBS, - | 0.0018 | -0.0040 – 0.0075 | 0.0013 | -0.0071 – 0.0097 | 0.0012 | -0.0068 – 0.0092 |
| 3^rd^ trimester |  |  |  |  |  |  |
| Spine BMC, g | -0.0239 | -0.2794 – 0.2316 | -0.0690 | -0.4155 – 0.2776 | 0.0179 | -0.3595 – 0.3954 |
| Spine BMD, g/cm^2^ | 0.0006 | -0.0044 – 0.0056 | 0.0010 | -0.0066 – 0.0087 | 0.0009 | -0.0063 – 0.0080 |
| Spine TBS, - | 0.0040 | -0.0020 – 0.0101 | 0.0035 | -0.0060 – 0.0130 | 0.0044 | -0.0038 – 0.0126 |
| **Model B** |  |  |  |  |  |  |
| 2^nd^ trimester |  |  |  |  |  |  |
| Spine BMC, g | -0.0174 | -0.4067 – 0.5860 | -0.0383 | -0.3804 – 0.3037 | **-0.4518** | **-0.8093 – -0.0942** |
| Spine BMD, g/cm^2^ | 0.0026 | -0.0023 – 0.0076 | 0.0050 | -0.0027 – 0.0127 | -0.0002 | -0.0075 – 0.0071 |
| Spine TBS, - | 0.0018 | -0.0042 – 0.0077 | 0.0004 | -0.0090 – 0.0098 | 0.0003 | -0.0083 – 0.0088 |
| 3^rd^ trimester |  |  |  |  |  |  |
| Spine BMC, g | -0.0429 | -0.3036 – 0.2179 | -0.0775 | -0.4555 – 0.3005 | -0.0814 | -0.4684 – 0.3055 |
| Spine BMD, g/cm^2^ | 0.0012 | -0.0042 – 0.0066 | 0.0009 | -0.0750 – 0.0094 | -0.0005 | -0.0078 – 0.0069 |
| Spine TBS, - | 0.0051 | -0.0014 – 0.0116 | 0.0038 | -0.0064 – 0.0140 | 0.0045 | -0.0042 – 0.0132 |
| **Model C** |  |  |  |  |  |  |
| 2^nd^ trimester |  |  |  |  |  |  |
| Spine BMC, g | -0.2106 | -0.4430 – 0.0022 | -0.1182 | -0.4664 – 0.2300 | **-0.4808** | **-0.8401 – -0.1216** |
| Spine BMD, g/cm^2^ | 0.0013 | -0.0035 – 0.0061 | 0.0027 | -0.0051 – 0.0106 | -0.0016 | -0.0081 – 0.0050 |
| Spine TBS, - | 0.0021 | -0.0038 – 0.0081 | 0.0031 | -0.0070 – 0.0132 | 0.0006 | -0.0082 – 0.0094 |
| 3^rd^ trimester |  |  |  |  |  |  |
| Spine BMC, g | -0.0361 | -0.2946 – 0.2224 | -0.0692 | -0.4304 – 0.2919 | -0.1254 | -0.5221 – 0.2713 |
| Spine BMD, g/cm^2^ | 0.0012 | -0.0040 – 0.0064 | 0.0003 | -0.0078 – 0.0085 | -0.0013 | -0.0080 – 0.0054 |
| Spine TBS, - | 0.0046 | -0.0020 – 0.0111 | 0.0046 | -0.0059 – 0.0151 | 0.0046 | -0.0044 – 0.0136 |
|  |  |  |  |  |  |  |

**Supplementary Table 1: Mean difference with 95% confidence interval in offspring bone measures at nine years of age per 0.1 µmol/L maternal serum retinol in 2^nd^ and 3^rd^ trimesters**

Model A: adjusted for child age and height (and sex in total column) Model B: model A + maternal age, parity, education, smoking during pregnancy and pre-pregnancy body mass index (kg/m^2^). Model C: models A + B + child serum vitamin 25(OH)D, child body weight, birthweight, gestational age at birth and child serum sample season.
