# Supplementary material for: Maternal vitamin A and D status in second and third trimester of pregnancy and bone mineral content in offspring at nine years of age
Source: Front Endocrinol (Lausanne). 2024 Jun 28;15:1417656. doi: 10.3389/fendo.2024.1417656 (PMC11239386; doi:10.3389/fendo.2024.1417656)
Supplement: Supplementary file 2 [file Table_2.docx]

Supplementary Material 2

# Maternal vitamin A and D in second and third trimester of pregnancy and offspring bone measures at nine years of age

Stunes, Astrid Kamilla*^1,2^, Mosti, Mats Peder^1,3^, Børsting, Torunn^2,4^, Thorsby, Per Medbøe^5^, Stafne, Signe Nilssen^4,6^, Syversen, Unni^1,7^.

^1^Clinical and Molecular Medicine, Faculty of Medicine and Health Sciences, Norwegian University of Science and Technology, Trondheim (NTNU), Norway

^2^Center for Oral Health Services and Research, Mid-Norway (TkMidt), Trondheim, Norway

^3^Department of Research and Development, Clinic of Substance Use and Addiction Medicine, St. Olavs University Hospital, Trondheim, Norway

^4^Department of Public Health and Nursing, Norwegian University of Science and Technology (NTNU), Trondheim, Norway

^5^Hormone Laboratory, Department of Medical Biochemistry, Oslo University Hospital, Aker, Oslo, Norway

^6^Clinic of Rehabilitation, St. Olavs Hospital, Trondheim University Hospital, Norway

^7^Department of Endocrinology, Clinic of Medicine, St. Olavs University Hospital, Trondheim, Norway

*** Correspondence:** Astrid Kamilla Stunes, kamilla.stunes@ntnu.no

**
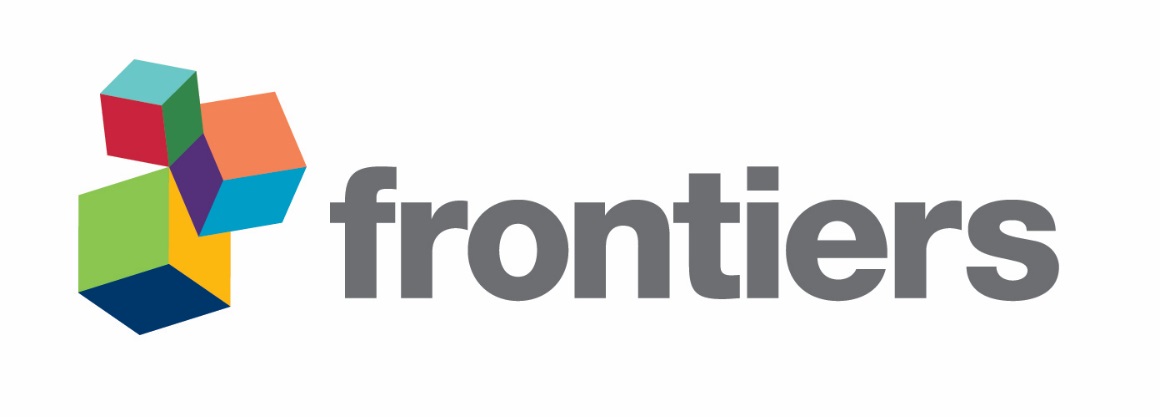
**

**Supplementary Table 2:** **Mean difference with 95% confidence interval in offspring bone measures at nine years of age per 10 nmol/L maternal serum vitamin D (25(OH)D) in 2^nd^ and 3^rd^ trimesters**

|  | **Total, n=119** | | **Girls, n=62 [52%]** | | **Boys, n=57 [48%]** | |
| --- | --- | --- | --- | --- | --- | --- |
| **Crude** | **β** | **95% CI** | **β** | **95% CI** | **β** | **95% CI** |
| 2^nd^ trimester |  |  |  |  |  |  |
| Spine BMC, g | -0.0098 | -0.2873 – 0.2676 | 0.0061 | -0.2870 – 0.4083 | -0.1306 | -0.5778 – 0.3167 |
| Spine BMD, g/cm^2^ | 0.0034 | -0.0016 – 0.0084 | 0.0014 | -0.0056 – 0.0085 | 0.0059 | -0.0014 – 0.0133 |
| Spine TBS, - | 0.0005 | -0.0056 – 0.0066 | -0.0018 | -0.0097 – 0.0061 | 0.0008 | -0.0070 – 0.0086 |
| 3^rd^ trimester |  |  |  |  |  |  |
| Spine BMC, g | -0.0187 | -0.2714 – 0.2339 | -0.0708 | -0.3860 – 0.2443 | 0.0043 | -0.3859 – 0.3945 |
| Spine BMD, g/cm^2^ | 0.0020 | -0.0025 – 0.0066 | -0.0001 | -0.0066 – 0.0063 | 0.0046 | -0.0018 – 0.0109 |
| Spine TBS, - | -0.0001 | -0.0056 – 0.0053 | -0.0052 | -0.0124 – 0.0021 | 0.0032 | -0.0034 – 0.0099 |
| **Model A** |  |  |  |  |  |  |
| 2^nd^ trimester |  |  |  |  |  |  |
| Spine BMC, g | -0.0843 | -0.3056 – 0.1369 | 0.0885 | -0.2032 – 0.3802 | -0.2711 | -0.6160 – 0.0737 |
| Spine BMD, g/cm^2^ | 0.0029 | -0.0017 – 0.0075 | 0.0019 | -0.0047 – 0.0085 | 0.0044 | -0.0023 – 0.0112 |
| Spine TBS, - | 0.0007 | -0.0062 – 0.0049 | -0.0009 | -0.0089 – 0.0070 | 0.0011 | -0.0067 – 0.0089 |
| 3^rd^ trimester |  |  |  |  |  |  |
| Spine BMC, g | -0.0115 | -0.2186 – 0.1955 | -0.0123 | -0.2910 – 0.2665 | -0.0681 | -0.3922 – 0.2560 |
| Spine BMD, g/cm^2^ | 0.0024 | -0.0018 – 0.0066 | 0.0004 | -0.0059 – 0.0068 | 0.0039 | -0.0022 – 0.0100 |
| Spine TBS, - | 0.0010 | -0.0061 – 0.0041 | -0.0049 | -0.0128 – 0.0030 | 0.0040 | -0.0031 – 0.0110 |
| **Model B** |  |  |  |  |  |  |
| 2^nd^ trimester |  |  |  |  |  |  |
| Spine BMC, g | -0.0958 | -0.3354 – 0.1438 | 0.1732 | -0.1479 – 0.4946 | -0.3025 | -0.6477 – 0.0426 |
| Spine BMD, g/cm^2^ | 0.0031 | -0.0020 – 0.0081 | 0.0034 | -0.0040 – 0.0011 | 0.0040 | -0.0029 – 0.0108 |
| Spine TBS, - | -0.0017 | -0.0077 – 0.0044 | 0.0003 | -0.0086 – 0.0096 | -0.0004 | -0.0084 – 0.0077 |
| 3^rd^ trimester |  |  |  |  |  |  |
| Spine BMC, g | -0.0396 | -0.2661 – 0.1869 | 0.0384 | -0.2848 – 0.3616 | -0.0826 | -0.4371 – 0.2718 |
| Spine BMD, g/cm^2^ | 0.0020 | -0.0027 – 0.0068 | 0.0023 | -0.0052 – 0.0098 | 0.0012 | -0.0068 – 0.0092 |
| Spine TBS, - | -0.0025 | -0.0082 – 0.0033 | -0.0065 | -0.0160 – 0.0030 | 0.0055 | -0.0024 – 0.0134 |
| **Model C** |  |  |  |  |  |  |
| 2^nd^ trimester |  |  |  |  |  |  |
| Spine BMC, g | -0.0937 | -0.3286 – 0.1412 | 0.2729 | -0.0397 – 0.5876 | -0.4190 | -0.8015 – 0.0363 |
| Spine BMD, g/cm^2^ | 0.0028 | -0.0020 – 0.0076 | 0.0061 | -0.0010 – 0.0133 | 0.0013 | -0.0056 – 0.0082 |
| Spine TBS, - | 0.0001 | -0.0058 – 0.0060 | -0.0012 | -0.0104 – 0.0081 | 0.0009 | -0.0082 – 0.0100 |
| 3^rd^ trimester |  |  |  |  |  |  |
| Spine BMC, g | -0.1040 | -0.3368 – 0.1288 | 0.0792 | -0.2500 – 0.4084 | -0.1927 | -0.6042 – 0.2187 |
| Spine BMD, g/cm^2^ | 0.0006 | -0.0042 – 0.0054 | 0.0041 | -0.0035 – 0.0117 | 0.0006 | -0.0057 – 0.0069 |
| Spine TBS, - | -0.0016 | -0.0077 – 0.0045 | -0.0083 | -0.0185 – 0.0019 | 0.0059 | -0.0030 – 0.0149 |
|  |  |  |  |  |  |  |

Model A: adjusted for child age and height (and sex in total column). Model B: model A + maternal age, parity, education, smoking during pregnancy, pre-pregnancy body mass index (kg/m^2^) and sample season. Model C: models A + B + child serum vitamin 25(OH)D, child body weight, birthweight, gestational age at birth and child serum sample season. Abbreviations: BMC=bone mineral content, BMD=bone mineral density, TBS=trabecular bone score.
